# Inhibition of indoleamine dioxygenase leads to better control of tuberculosis adjunctive to chemotherapy

**DOI:** 10.1172/jci.insight.163101

**Published:** 2023-01-24

**Authors:** Bindu Singh, Chivonne Moodley, Dhiraj K. Singh, Ruby A. Escobedo, Riti Sharan, Garima Arora, Shashank R. Ganatra, Vinay Shivanna, Olga Gonzalez, Shannan Hall-Ursone, Edward J. Dick, Deepak Kaushal, Xavier Alvarez, Smriti Mehra

**Affiliations:** Southwest National Primate Research Center, Texas Biomedical Research Institute, San Antonio, Texas, USA.

**Keywords:** Immunology, Infectious disease, Antigen presentation

## Abstract

The expression of indoleamine 2,3-dioxygenase (IDO), a robust immunosuppressant, is significantly induced in macaque tuberculosis (TB) granulomas, where it is expressed on IFN-responsive macrophages and myeloid-derived suppressor cells. IDO expression is also highly induced in human TB granulomas, and products of its activity are detected in patients with TB. In vivo blockade of IDO activity resulted in the reorganization of the granuloma with substantially greater T cells being recruited to the core of the lesions. This correlated with better immune control of TB and reduced lung *M. tuberculosis* burdens. To study if the IDO blockade strategy can be translated to a bona fide host-directed therapy in the clinical setting of TB, we studied the effect of IDO inhibitor 1-methyl-d-tryptophan adjunctive to suboptimal anti-TB chemotherapy. While two-thirds of controls and one-third of chemotherapy-treated animals progressed to active TB, inhibition of IDO adjunctive to the same therapy protected macaques from TB, as measured by clinical, radiological, and microbiological attributes. Although chemotherapy improved proliferative T cell responses, adjunctive inhibition of IDO further enhanced the recruitment of effector T cells to the lung. These results strongly suggest the possibility that IDO inhibition can be attempted adjunctive to anti-TB chemotherapy in clinical trials.

## Introduction

### Mycobacterium tuberculosis (M. tuberculosis), an important intracellular pathogen, causes approximately 1.8 million deaths every year through tuberculosis (TB) (1).

 Infection of human lungs with *M. tuberculosis* is characterized by a robust innate and adaptive immune response, which eventually leads to the formation of a pathological lesion called the granuloma, a hallmark of TB that influences the outcome of the infection (1). It is believed that the granuloma locally helps contain the infection, though the specific mechanisms by which the granuloma exerts immune control of *M. tuberculosis*, i.e., the spatial understanding of the granuloma function, have not been completely understood (2). The architecture and composition of the granuloma can, however, directly influence both the phenotype of the pathogen as well as the host immune response, thus affecting the disease outcome in many different ways (3). Single cell–based approaches are now being used to study gene and protein expression to better understand the TB lung (4–6). These techniques leverage both opportunistically available human granuloma samples as well as those from experimentally infected animal models (4, 7) and provide a much more detailed picture of granuloma gene expression, cellular composition, and function. Multiplexed imaging of the human TB granuloma recently revealed the highly immunosuppressed nature of the granuloma microenvironment (7) — human TB granulomas are depleted for IFN-γ^+^ cells but instead enriched for TGF-β, regulatory T cells (Tregs), and indoleamine 2,3-dioxygenase^+^ (IDO^+^) programmed cell death ligand 1^+^ myeloid cells. IDO is one of the most abundant proteins present in human TB granulomas (7). IDO catabolizes the essential amino acid tryptophan (Trp) to kynurenine (Kyn) (8) and exerts robust direct and indirect immunosuppressive effects on T cell activation (9).

Several studies in nonhuman primates have also reported that the TB granuloma is an immunosuppressive environment. Thus, while newly formed TB granulomas from rhesus macaques express a robust proinflammatory gene signature, this is rapidly reprogrammed by the expression of genes involved in tissue remodeling (10), coincident with the development of necrosis and hypoxia in these macaque lesions (11). This causes the pathogen to alter its in vivo phenotype dependent on the DosR regulon for intragranulomatous persistence (11, 12). Gideon et al. showed that an exceedingly small number of T cells from granulomas derived from cynomolgus macaques infected with *M. tuberculosis* respond with cytokine production after stimulation with *M. tuberculosis*–specific antigens, and few “multifunctional” T cells were observed (13). Our work in both rhesus and cynomolgus macaques showed that after *M. tuberculosis* infection, the expression of IDO is significantly induced in the myeloid layer of nonhuman primate TB granulomas (10, 14). Expression of IDO in response to *M. tuberculosis* infection is not limited to primate hosts but can be observed in murine models as well as in macrophages in vitro (15, 16). The induction of IDO expression in the lung occurs in a manner directly proportional to the burden of *M. tuberculosis* (16). We have since identified that IDO is primarily expressed on IFN-responsive, inflammatory, interstitial macrophages in the lungs of *M. tuberculosis*–infected macaques (4), as well as on immunosuppressive myeloid-derived suppressor cells (MDSCs) (17). A compendium of studies have since shown that IDO expression is highly induced in the human TB granuloma environment, and products of IDO-mediated Trp catabolism are detected in the plasma, sera, and urine of patients with active TB, including multidrug-resistant tuberculosis (MDR-TB) as well as TB/HIV, in cohorts from various regions of the world (18–20). Taken together, these results from animal models of TB as well as patients unequivocally show that the expression of IDO, a potent immunosuppressor of T cell activity, is induced in macrophages infected with *M. tuberculosis* and that this expression can be clearly observed in granulomas, which are a highly immunosuppressed environment.

In the case of many intracellular pathogenic organisms, e.g., *Chlamydia*, *Leishmania*, *Coxiella* and *Listeria*, host-mediated catabolism of the essential amino acid Trp, initiated by the activation of the rate-limiting enzyme, IDO, represents an effective means of innate immune control (21–24). Granuloma-resident *M. tuberculosis* is, however, able to synthesize Trp (12, 25, 26). Unfortunately, therefore, the host’s strategy to deplete Trp is ineffective during *M. tuberculosis* infection, and furthermore, downstream Kyn metabolites of this pathway impair phagosome/lysosome fusion and autophagy (27), processes that serve to kill intracellular *M. tuberculosis*. Furthermore, downstream metabolites of the IDO pathway serve to impair the function of CD4^+^ T cells, via expanding Tregs and MDSCs (17, 28). Coupled with the lack of Trp for proliferating T cells, these mechanisms create an immunosuppressive environment conducive to the persistence of *M. tuberculosis*. Thus, the IDO pathway is ineffective and actually deleterious during *M. tuberculosis* infection. Since IDO is a powerful suppressant of T cell function, this ineffectiveness of the IDO pathway to control *M. tuberculosis* in vivo, coupled with the strong induction of IDO in TB granulomas, together suggest that blockade of IDO activity in vivo may also serve as an attractive host-directed therapy (HDT) target for TB. Our group has developed a macaque model of *M. tuberculosis* infection via the natural, aerosol route of exposure (11, 29, 30). Based on the choice of strain and dose of infection, macaques either develop immune control of *M. tuberculosis* similar to latent tuberculosis infection (LTBI) (11, 31) or progress to pulmonary TB (29, 30). Coinfection of controllers with simian immunodeficiency virus SIVmac239 results in the reactivation of LTBI (31–34). Furthermore, in our model, reactivation strongly correlates with the presence of chronic immune activation in lungs (35). We therefore investigated the ability of an IDO inhibitor to provide antimicrobial activity as well as enhance adaptive and innate immunity in a macaque model of active TB. In vivo blockade of IDO activity, using monotherapy with 1-methyl-d-tryptophan (D1MT), during *M. tuberculosis* infection leading to TB was indeed beneficial to the host (16). However, in real life, IDO inhibitors are unlikely to be used as monotherapy; furthermore, our model of active TB, while beneficial for the evaluation of vaccines and therapeutics, requires exposure to a nonphysiological high dose of *M. tuberculosis* for infection and may not represent a real-life situation. We therefore devised an experiment where we tested the effectiveness of D1MT to adjunctively enhance the effectiveness of a deliberately suboptimal chemotherapeutic regimen against TB, in a model where macaques were infected with a dose of drug-sensitive *M. tuberculosis* CDC1551, a low-virulence strain, such that about 50% of the animals develop active TB (11). Our results suggest that IDO inhibition can indeed improve immune responses and adjunctively enhance the chemotherapeutic potential of anti-TB therapy.

## Results

### M. tuberculosis infection and inclusion of animals in different groups of treatment.

**Figure 1:**
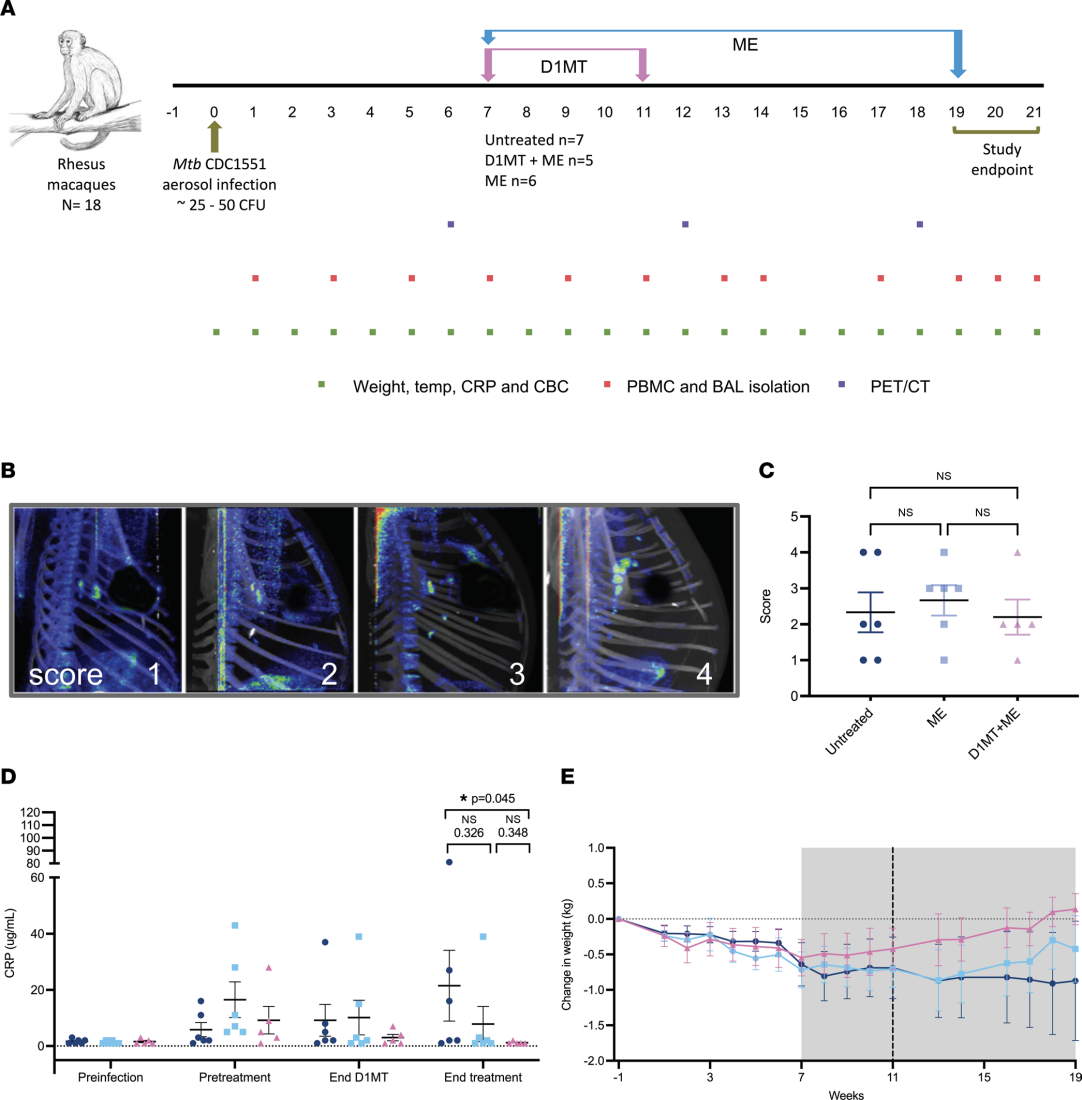
D1MT and moxifloxacin and ethambutol treatment alone or in conjunction has no distinct effect on clinical correlates of TB disease. (**A**) Shown is the study timeline. Eighteen Indian origin rhesus macaques (RMs) were infected with medium-high dose (~25–50 CFU) of *M*. *tuberculosis* CDC1551 via aerosol route. The RMs developed TB disease around week 6 postinfection, as confirmed by PET/CT scans, and were subsequently divided into 3 groups: untreated, ME, and ME/D1MT. The treatment started at week 7 postinfection and continued through week 19 postinfection. The RMs were then euthanized, which marked the endpoint of the study. Complete blood count (CBC), CRP, and changes in weight and temperature were monitored weekly, whereas blood and bronchoalveolar lavage (BAL) were collected every 2 weeks for performing immunological assays, except for week 15, as we shifted the scheduled week 15 time point to week 14 because of the winter break. Week 19 through week 21, the necropsies took place, and we collected BAL and blood before euthanizing individual macaques. PET/CT scans were done at week 6, week 12, and week 18 after *M*. *tuberculosis* infection. (**B**) Representative PET/CT images of the *M*. *tuberculosis*–infected macaques at week 6 postinfection depicting varying degrees of lesions in the lung, used for unbiased classification of the macaques into 3 experimental groups. The RMs were designated scores 1–4. (**C**) Graph depicting the distribution of macaques based on the ranks of severity of *M*. *tuberculosis* infection by lesion fluorodeoxyglucose F18 (FDG) uptake (PET/CT scores) at week 6 to evenly divide into 3 groups. (**D**) Graphical representation of average serum CRP levels (μg/mL) at experimental time points. (**E**) Graph depicting the mean change in weight (kg) with respect to the baseline values among the 3 groups of macaques over the course of the study. Gray area represents treatment phase, whereas black dotted line shows end of D1MT treatment time point. *P* values are indicated above the plots as obtained from 1-way ANOVA (**C**) and Mann-Whitney test (**D**). Data are represented as mean ± SEM.

A total of 18 Indian origin rhesus macaques (RMs) were infected with approximately 25–50 CFU *M. tuberculosis* CDC1551 (Figure 1A). We have previously shown that this dose of infection with a low-virulence strain of *M. tuberculosis* results in the progression of approximately 50% of the RMs to active TB over 2–4 months. The experimental design illustration (Figure 1A) includes details about the infection, treatment groups, the period of treatment, and procedures performed. As indicated, PET/CT scans were performed at a preinfection time point, as well as at weeks 6, 12, and 18 (or earlier if necessary, at the endpoint). RMs were assigned to 1 of 3 groups based on the week 6 PET/CT scores. The 3 groups were untreated control group receiving no treatment following *M. tuberculosis* infection; ME treatment group receiving moxifloxacin and ethambutol (M, 10 mg/kg; E, 20 mg/kg) regimen following *M. tuberculosis* infection between weeks 7 and 19, i.e., for 12 weeks; and ME/D1MT treatment group receiving moxifloxacin and ethambutol (M, 10 mg/kg; E, 20 mg/kg) regimen for 12 weeks as described above along with concurrent treatment with 45 mg/kg IDO inhibitor D1MT daily, at the beginning of the chemotherapy, between weeks 7 and 11. Because of our choice of the dose of *M. tuberculosis* for aerosol infection, some animals developed disease, as measured by high PET/CT scores (scores > 3), while others showed signs of TB (scores 2–3), and yet others had minimal evidence of TB (scores 1) (Figure 1, B and C). This is unlike aerosol infection with a high dose of *M. tuberculosis*, where all animals rapidly develop active TB (16), or with a very low dose of *M. tuberculosis*, where nearly all animals develop LTBI (33, 34). PET/CT scores generated in a blinded fashion from macaques imaged at week 6, before the initiation of any therapy, are shown in Figure 1C. The macaques were assigned to 1 of the 3 groups such that each group had an even distribution of scores and that no bias was introduced due to heterogeneity in disease levels. As is evident from the graph shown in Figure 1C, there were no differences in the PET/CT scores of the macaques in the 3 groups before initiation of treatment.

### Treatment with ME (alone as well as in combination with D1MT) results in improvement of clinical parameters.

Both groups of RMs that received ME treatment (ME and ME/D1MT) harbored significantly lower clinical levels of TB disease as measured by serum C-reactive protein (CRP) levels (Figure 1D) and percentage change in body weights (Figure 1E). Serum CRP levels in the macaques of the ME/D1MT group were found to be significantly reduced after 12 weeks of treatment as compared with untreated group (Figure 1D). As is evident from the line graph shown in Figure 1E, upon the treatment initiation, all animals initially lost weight. Upon the initiation of treatment, the ME/D1MT group started gaining weight, whereas the ME-only and the untreated group continued to lose weight. Around week 12 (after 5 weeks of ME treatment), the ME group also started gaining weight but to a lesser extent as compared with the ME/D1MT group. However, the control group continued to exhibit weight loss till the endpoint, such that on an average every RM in this group had lost an average of more than 0.5 kg in body weight compared with preinfection levels (Figure 1E). The graph depicting the change in weight at the endpoint (or week 19) is shown in Supplemental Figure 1A; supplemental material available online with this article; https://doi.org/10.1172/jci.insight.163101DS1 No notable differences were observed in change in temperature among the 3 groups (Supplemental Figure 1B).

### Inclusion of D1MT adjunctive to ME results in the inhibition of IDO enzymatic activity in a model of heterogeneous TB progression.

**Figure 2:**
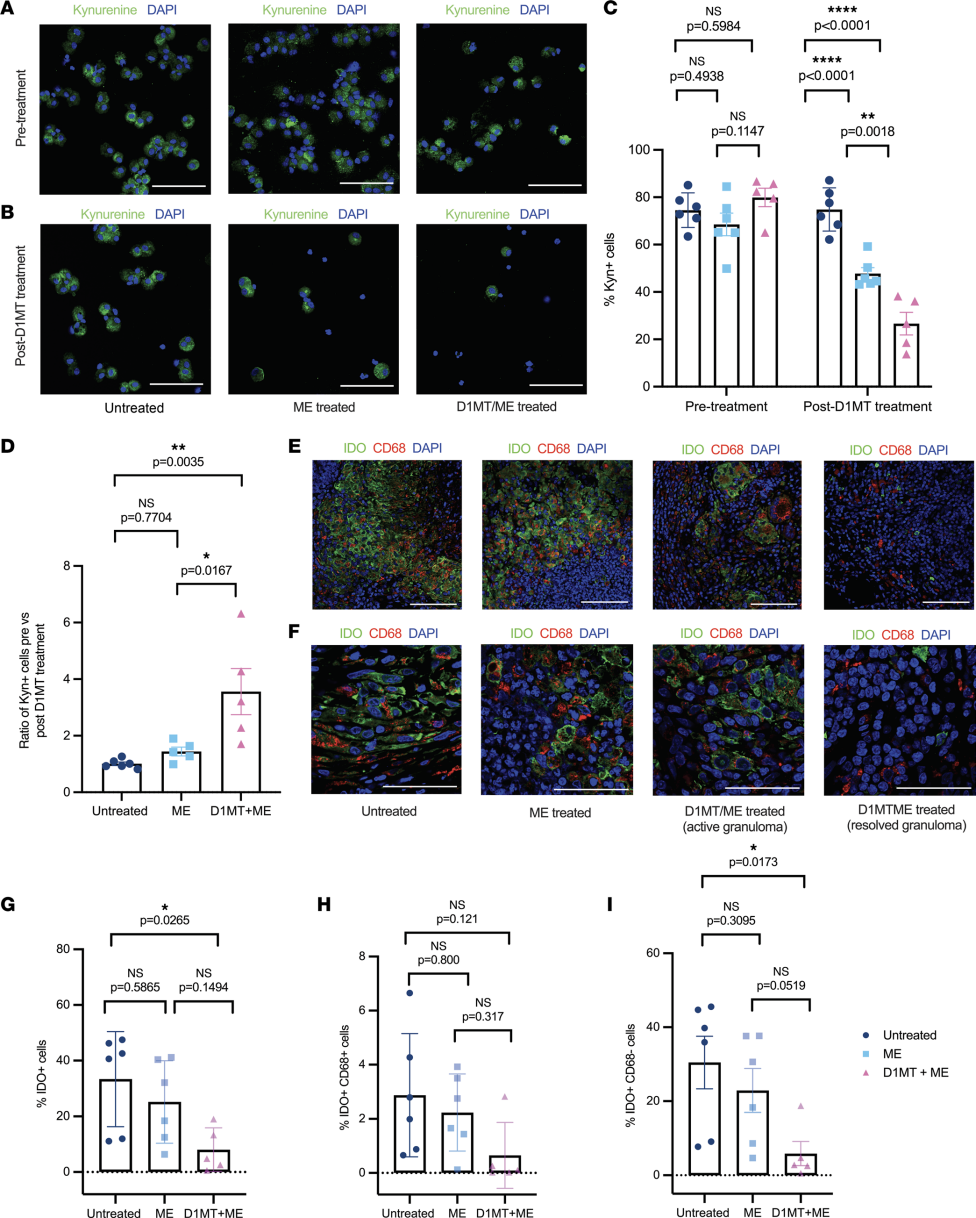
Inclusion of D1MT adjunctive to ME results in the inhibition of IDO enzymatic activity. (**A–D**) Immunofluorescence was performed on BAL cells collected at week 7 postinfection (pre-treatment) and week 11 postinfection (post-D1MT treatment) for kynurenine (Kyn). Representative confocal images showing Kyn (green) and nucleus (blue) in BAL cells prior to the start of the treatment (week 7) (**A**) and at the end of D1MT treatment (week 11) (**B**). Twelve random fields per animal in all 3 groups were captured using Zeiss LSM 800 confocal microscope and quantified using ImageJ (Fiji) software. (**C**) Graph showing the percentages of Kyn-positive cells present in BAL before and after D1MT treatment. (**D**) Graphical representation of the ratio of Kyn-positive cells before versus after D1MT treatment among the 3 groups. The ME/D1MT group depicted significantly higher ratios compared with the other 2 groups. (**E–I**) The lung sections obtained at necropsy were stained for pan-macrophage marker CD68, IDO, and DAPI (nucleus). The representative multilabel confocal images of the lung granulomas of the untreated, ME-treated, and ME/D1MT-treated macaques as well as resolved granuloma in ME/D1MT-treated macaques depicting CD68 (red), IDO (green), and nucleus (blue) captured at 20× (**E**) and 63× (**F**) original magnification. These images were captured using Zeiss LSM 800 confocal microscope. The whole tissue sections stained with CD68, IDO, and DAPI were then scanned using the slide scanner (Zeiss Axio Scan Z1) and were subsequently quantified using HALO analysis software for IDO expression by macrophages. (**G**) Graph showing the percentages of IDO-expressing cells or IDO^+^ cells, (**H**) IDO-expressing macrophages or IDO^+^CD68^+^ cells, and (**I**) IDO-expressing cells other than macrophages or IDO^+^CD68^–^ in the 3 groups. Scale bars, 100 μm (original magnification, 20×; **A**, **B**, and **E**) and 50 μm (original magnification, 63×; **F**). *P* values are indicated above the plots as obtained from 2-way ANOVA (**C**) and 1-way ANOVA (**D**, **G**, **H**, and **I**) with Tukey’s multiple-comparison test. Data are represented as mean ± SEM.

We measured IDO enzymatic activity by detecting Kyn (the major end product of the IDO pathway) by immunofluorescence staining in BAL-derived cells from the 3 groups of RMs collected at pretreatment time point (week 7) and after D1MT and ME treatment for 4 weeks (week 11). Representative images of BAL cells stained with Kyn antibody are shown in Figure 2, A and B. Quantification of Kyn-positive cells in BAL revealed that before the initiation of treatment (i.e., at week 7), all the macaques had high levels of Kyn in BAL, which accounted for the presence of 65%–80% Kyn-positive cells, which was comparable among the 3 groups. However, after 4 weeks of treatment, both the ME/D1MT and the ME groups exhibited a significant reduction in the percentage of Kyn-positive cells as compared with the untreated group as shown in Figure 2C. The reduction was more evident in the ME/D1MT group, with the presence of only approximately 26% Kyn-positive cells compared with approximately 48% in the ME group. Inclusion of D1MT for 4 weeks, therefore, resulted in significant reduction of IDO activity. Although the differences between the 2 treated groups were highly significant, the percentages of Kyn-positive cells were also significantly reduced in both treatment groups as compared with the untreated one. We also calculated the ratio of Kyn-positive cells pretreatment versus posttreatment, where we observed significantly high values in the ME/D1MT group as compared with untreated as well as ME-only groups as depicted in Figure 2D.

IDO levels in lung (at endpoint) and BAL cells (before and after D1MT treatment) were also assessed by quantitative reverse transcription PCR (qRT-PCR). We could see some reduction in IDO levels in lungs (Supplemental Figure 2A), though the difference was statistically not significant. However, no marked differences were observed in BAL cells before and after D1MT treatment (Supplemental Figure 2E). The levels of a few other gene transcripts, including IDO2, IFN-γ, and IFN-β, were also quantified by qRT-PCR, with no notable differences (Supplemental Figure 2, B–D and F–H). We next performed multi-labeled immunohistochemistry by staining lung tissues for IDO expression in macrophages, using anti-IDO and anti-CD68 antibodies. The representative confocal images from untreated, ME only–treated, and ME/D1MT-treated macaques are shown in Figure 2E (20× original magnification) and Figure 2F (63× original magnification). The third panel of Figure 2E shows the IDO expression in granuloma present in the ME/D1MT-treated animals, and the fourth panel depicts IDO expression in a resolved granuloma. The latter was a more common occurrence in ME/D1MT-treated RMs. The images were analyzed using HALO analysis software in a blinded manner to quantify the percentage of IDO-expressing cells in lungs (Figure 2G) as well as IDO-expressing macrophages (Figure 2H) and other cell types (Figure 2I). We observed significant decrease in IDO-positive cells in the ME/D1MT-treated group as compared with the untreated group (Figure 2G). However, no notable difference was observed in the percentages of IDO-expressing cells in the ME/D1MT group in comparison to the ME-only group. Interestingly, no significant differences were observed between the ME and untreated groups. The ME/D1MT group had the least bacillary burden and pathology of all 3 groups tested and was therefore likely to harbor less cellular infiltration. This could explain why statistically significant differences were not obtained between the 2 treatment groups. However, no significant differences were observed in the fraction of IDO-expressing macrophages in the lungs of the 3 groups of RMs (Figure 2H), but IDO-expressing cell types other than macrophages were significantly higher in the untreated group in comparison with the ME/D1MT group (Figure 2I).

### Inclusion of D1MT adjunctive to ME treatment results in better control of M.

**Figure 3:**
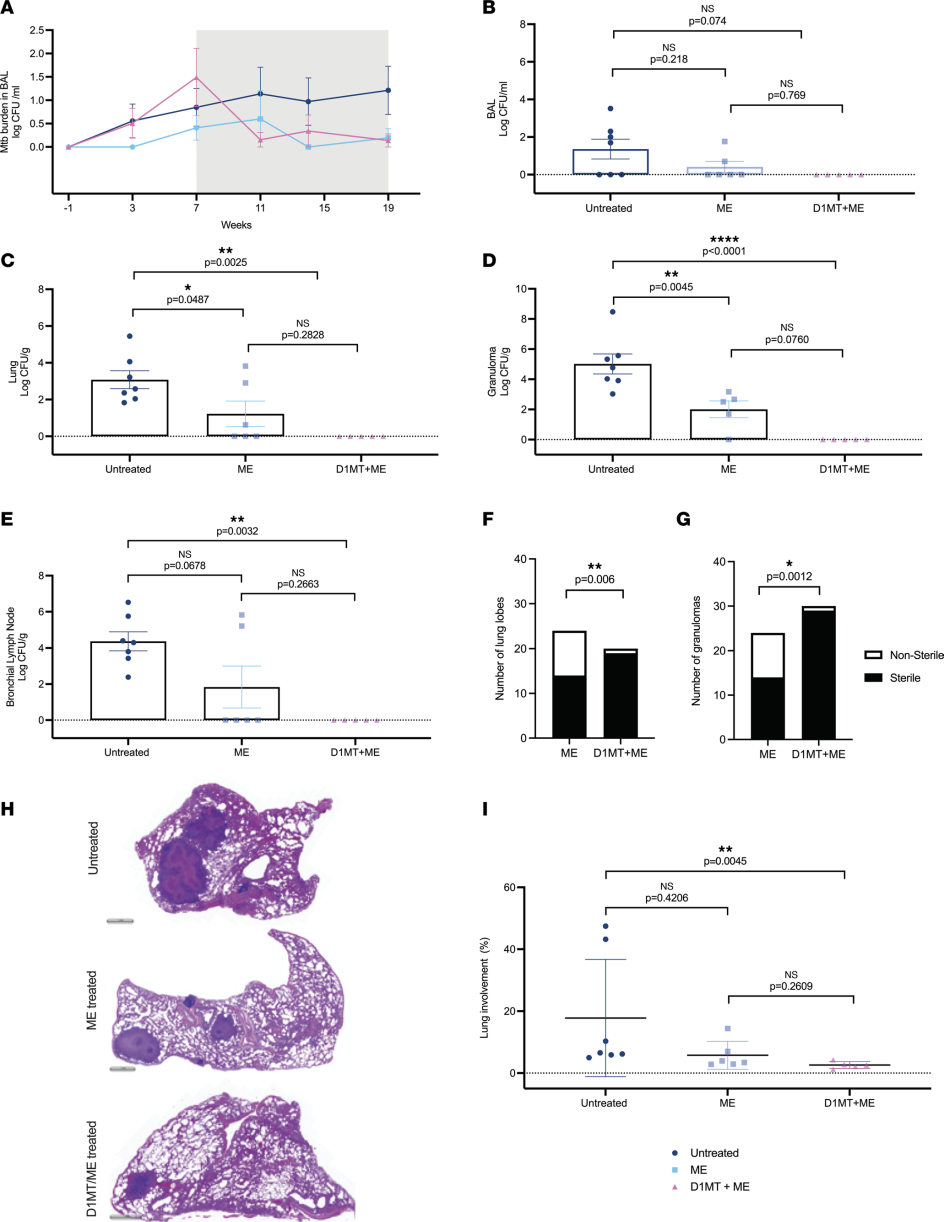
Inclusion of D1MT adjunctive to ME treatment results in better control of *M. tuberculosis* infection with complete clearance of bacilli. (**A–E**) *M*. *tuberculosis* burdens were assessed in BAL collected at various time points during the study timeline and at the endpoint. (**A**) Graph shows log *M*. *tuberculosis* CFU/mL of BAL in the 3 study groups at time points ranging from preinfection to the end of treatment. Gray area represents whole treatment period, whereas black dotted line shows end of D1MT treatment time point. Graphical representations of log CFU/mL of BAL (**B**), log CFU/g of lung (**C**), log CFU/g of granulomas (**D**), and log CFU/g of bronchial lymph nodes (**E**) obtained at endpoint/necropsy from macaques belonging to the 3 study groups. D1MT treatment led to complete clearance of *M*. *tuberculosis* from tissues. Further, lung tissues and granulomas obtained were assessed for sterility by using Fischer’s sterility test, to determine the sterile and nonsterile (in terms of *M*. *tuberculosis* presence) lung lobes and granulomas in ME and ME/D1MT groups. The graphs (**F** and **G**) depict the number of sterile (samples recording 0 CFUs) lung lobes and granulomas, respectively, among the 2 selected groups. H&E staining was performed on lung tissues obtained from all RMs at the endpoint. (**H**) Shown are representative images of H&E staining of lung from each group. Scale bars, 1 mm. H&E-stained lung sections were then scanned using Zeiss Axio Scan Z1 slide scanner and were used to quantify the lung area involved in inflammation or granulomatous lesions using HALO analysis software. (**I**) The graph depicts differences in the percentage lung involvement among the groups. For accurate depiction of 0 on log scale, a numeric value of 1 CFU was added to all CFU values before transforming them into log values, so that we have a value for 0 CFU or nondetectable CFU. *P* values are indicated above the plots as obtained from 1-way ANOVA (**B–E**) with Tukey’s multiple-comparison test, contingency χ^2^ (and Fisher’s exact) test (**F** and **G**) to compare number of sterile (samples recording 0 CFUs) lung lobes (**F**) and granulomas (**G**) between the groups using 2×2 contingency tables, and Kruskal-Wallis test (**I**). Data are represented as mean ± SEM.

*tuberculosis infection with complete clearance of bacilli*. *M. tuberculosis* burden longitudinally assessed in BAL at various points of the study timeline (Figure 3A), and at the endpoint in the same sample (Figure 3B), showed no marked differences among the 3 groups. A more prominent decrease in the ME/D1MT group was also observed at the endpoint relative to the ME group (Figure 3B). Significant reduction was observed in *M. tuberculosis* levels at the endpoint in lungs in the ME/D1MT group versus untreated group, with no significant reduction in the ME/D1MT group as compared with ME only (Figure 3C). *M. tuberculosis* burden assessment in lung granulomas also showed a significant reduction in the treated groups relative to the untreated group (Figure 3D). We also determined CFUs in mesenteric lymph node, liver, spleen, and kidney. We observed that the macaques in the ME/D1MT group had completely sterile organs, with few detectable CFUs in untreated and ME groups, but no statistical differences among the 3 groups were found (Supplemental Figure 1). CFU results from the lung-draining lymph nodes also depicted a significant decrease in bacterial load in the ME/D1MT but not in the ME treatment group relative to the untreated one (Figure 3E). These data suggest that D1MT in adjunct to ME therapy is more efficient in reducing/sterilizing *M. tuberculosis* than the suboptimal therapy with ME alone, whereas ME alone, while reducing *M. tuberculosis* burdens in lungs and granulomas of RMs, fails to sterilize the tissues completely (Figure 3, B–E). The extent of sterile lobes in the lungs (Figure 3F) and the number of individual granulomas derived from the lungs that were sterile (Figure 3G) were significantly higher in ME/D1MT-treated lungs and granulomas relative to the ME-only group.

H&E staining (Figure 3H) and quantification of affected lung area (marked by inflammation and presence of lesions) depicted a marked decrease in percentage lung involvement in the ME/D1MT group with respect to the untreated group as well as reduced lung involvement when compared with the ME-only group (Figure 3I). Analysis of gross lung pathology at the endpoint was congruent with these results, with the greatest percentage of pathology being observed in the control group, followed by the ME group and then the ME/D1MT group, which exhibited the least amount of lung pathology (Supplemental Figure 1, D–F). The representative H&E images from each macaque are depicted in Supplemental Figure 3.

### Validation of the superior effectiveness of D1MT in controlling M. tuberculosis infection adjunctive to ME in RMs, by PET/CT radiology.

TB pathogenesis and efficacy of ME and ME/D1MT prophylaxis regimens were examined using PET/CT scans (36). We used PET/CT as the primary correlate of progression of *M. tuberculosis* infection in the lungs of RMs. As described earlier, week 6 PET/CT images and scores were used to assign animals to different groups so that each group contained RMs with comparable disease progression at that time point. We also performed PET/CT imaging at week 12 (which was at the end of the D1MT treatment in the ME/D1MT group and one-third of the way in the 12-week ME treatment group). Finally, PET/CT imaging/analysis was performed at week 18 or endpoint, if earlier.

**Figure 4:**
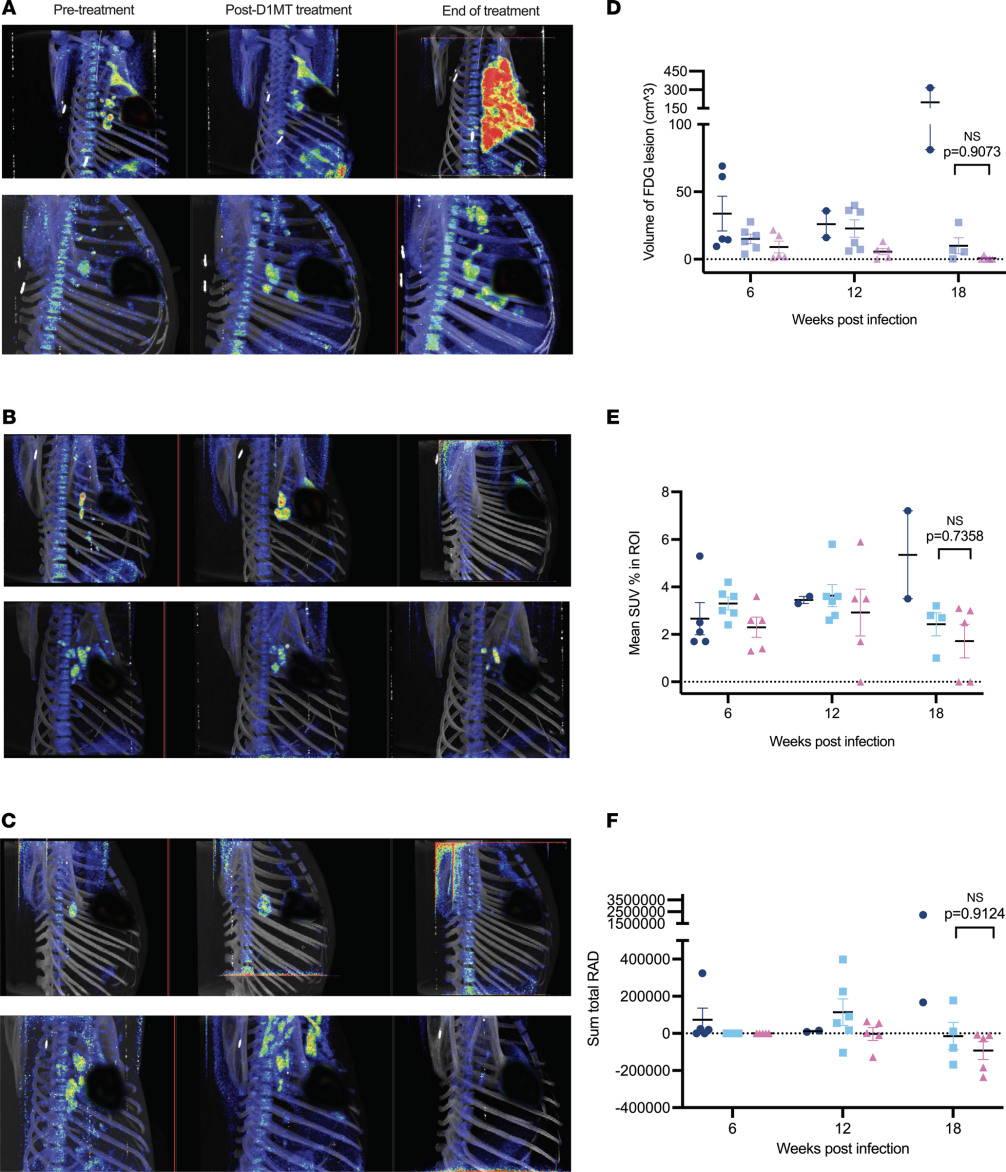
Superior effectiveness of D1MT in controlling *M. tuberculosis* infection adjunctive to ME confirmed by PET/CT radiology. PET/CT scans were performed at week 6 (pretreatment), week 12 (after D1MT treatment), and week 19/endpoint (end of treatment). (**A**–**C**) Shown are the PET/CT images from 2 macaques each from untreated (**A**), ME only–treated (**B**), and ME/D1MT-treated (**C**) groups at the abovementioned time points. The parameters measuring the activity in the lungs of macaques, including volume of FDG lesions (**D**), percentage mean SUV in regions of interest (ROI) (**E**), and sum total of unit of absorbed radiation dose (RAD) (**F**), are shown here. *P* values are indicated above the plots as obtained from 2-way ANOVA (**D**–**F**) with Tukey’s multiple-comparison test. Data are represented as mean ± SEM.

All the macaques (18/18) in the study had clear lungs prior to infection and focal nodular lung opacities at week 6 (pretreatment). The average PET/CT score in each group was 2.67, 2.33, and 2.2, respectively, for the control, ME, and ME/D1MT groups. All 18 animals had mild to moderate lymph node enlargement by 6 weeks after aerosol *M. tuberculosis* infection. The 18F-fluorodeoxyglucose (FDG) scans performed at either 12 or 18 weeks postinfection or at endpoint, if earlier, clearly revealed both the presence of persistent infection in the controls (Figure 4A) and the partial effectiveness of the ME regimen (Figure 4B) at the completion of the treatment; furthermore, these results exhibit the enhanced effectiveness of the ME regimen when D1MT was included in the treatment for the first 4 weeks (Figure 4C). Scans in the treated groups displayed no new lung lesions, while the previously reported lung lesions were resolved, although to a higher level in the ME/D1MT over ME group, i.e., no increase in lesion volume and no increase in FDG uptake (Figure 4D). In contrast, control animals displayed an increase in the size of lung lesions and increased FDG standardized uptake value (SUV) (Figure 4, D and E). All control animals showed TB and further progression of lung TB pathology including involvement of multiple lung lobes, with consolidation, lobar collapse, cavitary lesions, and massive mediastinal lymph node enlargement. The number and volume of lung lesions (Figure 4D), mean SUV (Figure 4E), and total lung activity (Figure 4F) of control animals was each higher compared with 2 treatment groups after treatment completion. Importantly, these values were lower for the ME/D1MT group relative to the ME-only group, clearly suggesting an advantage due to IDO blockade (Figure 4, D–F); however, the differences were not statistically significant between the 2 treatment groups.

Our results suggest that the extent of TB pathology and disease increased over time in control, untreated RMs (Figure 4A), as compared with ME only–treated (Figure 4B) and ME/D1MT-treated groups (Figure 4C), and that these groups of animals harbored different levels of pulmonary disease as measured by the various radiology attributes (Figure 4, D–F). *M. tuberculosis* infection led to development of varying degrees of active TB in untreated animals, as demonstrated by the presence of numerous granulomatous lesions by CT scans (Figure 4A) and the increased volume of FDG lesions (Figure 4D). All untreated, *M. tuberculosis*–infected animals had substantial evidence of granulomatous lesions (Figure 4A). Furthermore, the RMs in the 2 treatment groups did not demonstrate the presence of a significant number of lesions at week 12/endpoint or 18/endpoint (Figure 4, B and C). RMs in the control group showed gradual progression in TB pathology over time with multiple new lung lesions and an increase in size of previously emergent nodular lung lesions.

### Superior control of M. tuberculosis infection by ME adjunctive to D1MT is accompanied by improved T cell immune responses.

**Figure 5:**
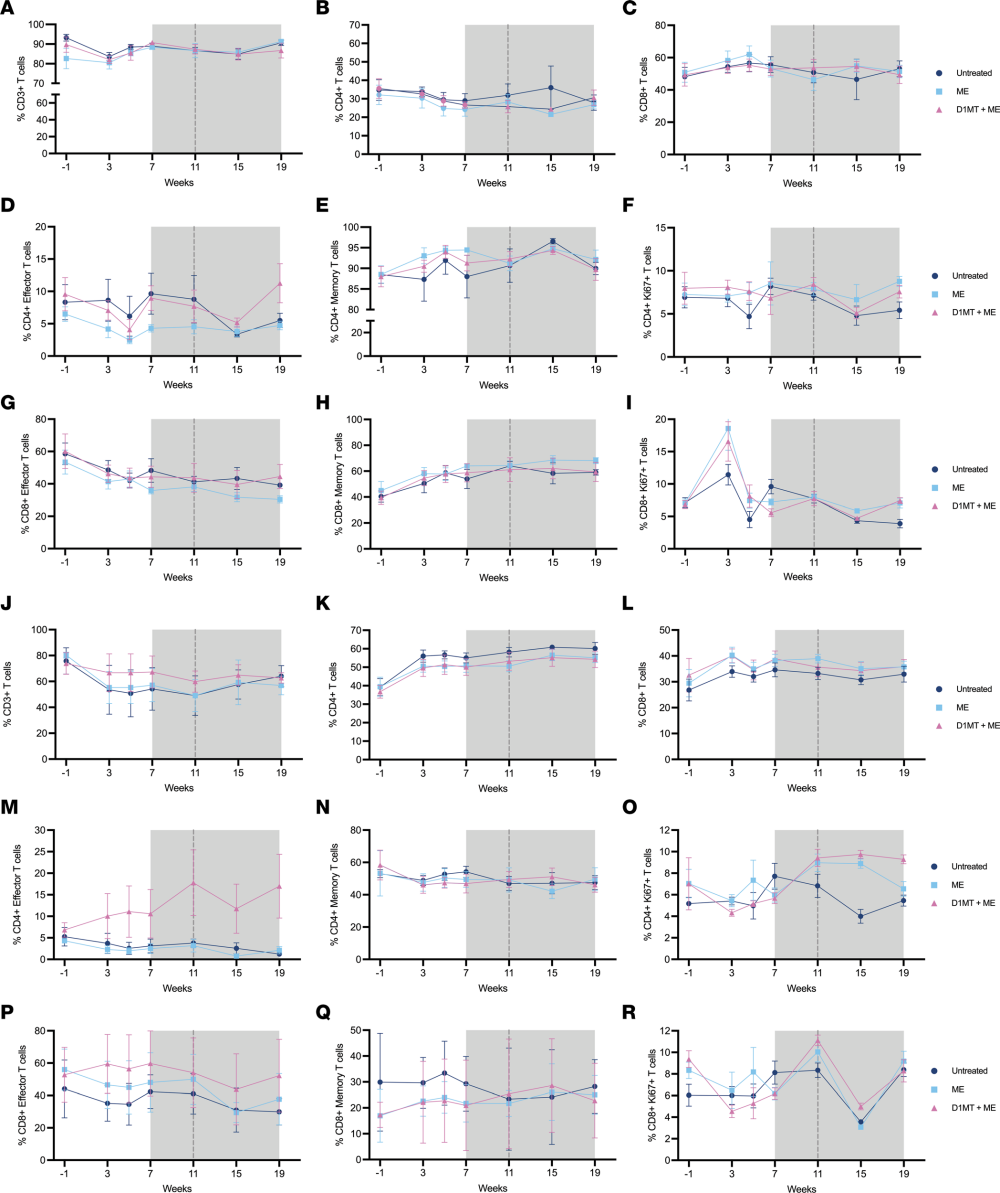
Improved T cell immune responses observed in treatment groups. T cell function and activation were studied by multiparameter flow cytometry on BAL and PBMCs obtained at various time points during the study. Shown are the data from flow cytometry experiments depicting the percentages of total CD3^+^ T cells (**A**), CD4^+^ T cells (**B**), CD8^+^ T cells (**C**), CD4^+^ effector T cells (**D**), CD4^+^ memory T cells (**E**), CD4^+^Ki67^+^ T cells (**F**), CD8^+^ effector T cells (**G**), CD8^+^ memory T cells (**H**), and CD8^+^Ki67^+^ T cells (**I**) in BAL and the percentages of total CD3^+^ T cells (**J**), CD4^+^ T cells (**K**), CD8^+^ T cells (**L**), CD4^+^ effector T cells (**M**), CD4^+^ memory T cells (**N**), CD4^+^Ki67^+^ T cells (**O**), CD8^+^ effector T cells (**P**), CD8^+^ memory T cells (**Q**), and CD8^+^Ki67^+^ T cells (**R**) in PBMCs. Gray area represents the whole treatment period, whereas black dotted line shows end of D1MT treatment time point. Data are represented as mean ± SEM.

**Figure 6:**
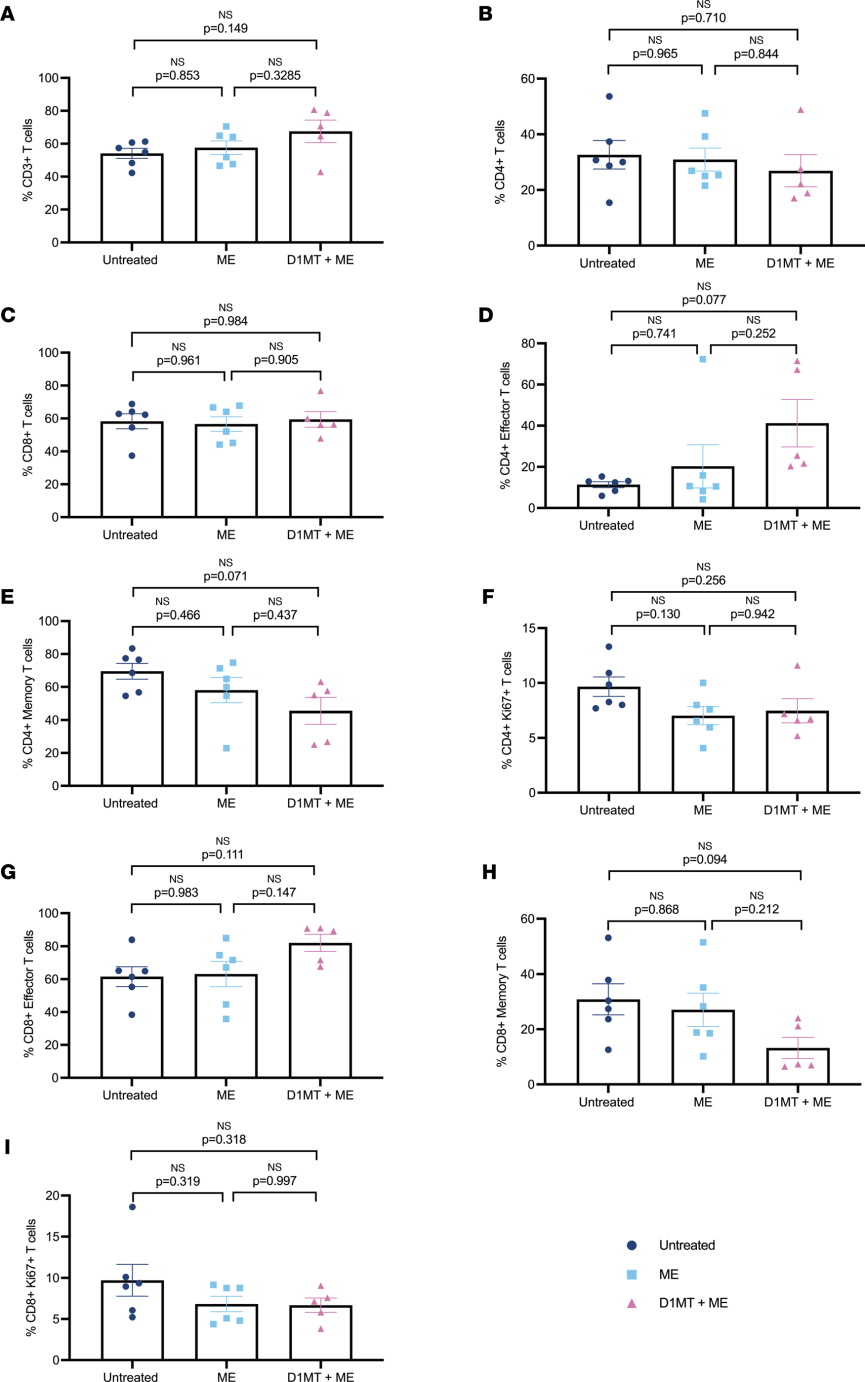
No significant differences are observed for total T cell counts in lungs of the 3 study groups. Lungs at the endpoint were processed to obtain single cells, which were subsequently stained for T cells and other functional markers. (**A–I**) The graphs illustrate the percentages of total CD3^+^ T cells (**A**), CD4^+^ T cells (**B**), CD8^+^ T cells (**C**), CD4^+^ effector T cells (**D**), CD4^+^ memory T cells (**E**), CD4^+^Ki67^+^ T cells (**F**), CD8^+^ effector T cells (**G**), CD8^+^ memory T cells (**H**), and CD8^+^Ki67^+^ T cells (**I**) in lungs at the endpoint. *P* values are indicated above the plots as obtained from 1-way ANOVA with Tukey’s multiple-comparison test. Data are represented as mean ± SEM.

We have shown earlier that D1MT treatment of RMs improved granuloma-specific immune responses. Here, we studied if overall T cell activation, proliferation, and recruitment as well as their antigen specificity was improved in the ME/D1MT relative to ME and control groups. Figure 5 shows the percentages of total CD3^+^ cells, CD4^+^ cells, CD8^+^ cells, CD4^+^ effector T cells, CD4^+^ memory T cells, CD4^+^ Ki67^+^ T cells, CD8^+^ effector T cells, CD8^+^ memory T cells, and CD8^+^Ki67^+^ T cells in BAL (Figure 5, A–I) and PBMCs (Figure 5, J–R) of the 3 groups at various time points of the study. It was only at the endpoint (Supplemental Figure 5) that we observed higher percentages of CD4^+^ effector T cells in BAL (Figure 5D and Supplemental Figure 5D) of ME/D1MT macaques in comparison with ME only–treated and untreated groups, with no changes observed in CD3^+^ T cells (Figure 5, A and J), CD4^+^ T cells (Figure 5, B and K), CD8^+^ T cells (Figure 5, C and L), CD4^+^ memory T cells (Figure 5, E and N), CD8^+^ effector T cells (Figure 5, G and P), and CD8^+^ memory T cells (Figure 5, H and Q). Both the treated groups exhibited significantly higher percentages of proliferative CD4^+^Ki67^+^ T cells (Figure 5F and Supplemental Figure 5F) and CD8^+^Ki67^+^ T cells (Figure 5I and Supplemental Figure 5I) in BAL relative to the untreated group. Flow cytometry analysis of T cells in lungs at the endpoint showed no significant changes in the overall percentages of CD3^+^, CD4^+^, and CD8^+^ cells; CD4^+^ effector T cells; CD4^+^ memory T cells; CD8^+^ effector T cells; CD8^+^ memory T cells; CD4^+^Ki67^+^ T cells; and CD8^+^Ki67^+^ T cells (Figure 6, A–I) among the 3 groups.

## Discussion

HDTs are an exciting new area of research in the field of TB (37–42). HDTs seek to modulate specific host immune pathways, including those that affect inflammation and immunopathology, to limit *M. tuberculosis* infection, persistence, reactivation, dissemination, and resulting pathology (37–42). Interest in HDT for TB is driven by the length of conventional TB therapy regimens and the desire to shorten them to increase compliance, thus reducing the incidence of MDR-TB. The concept of treating TB with adjunctive HDT also incorporates increasing knowledge that productive immune responses are subverted during pulmonary TB. A number of preclinical studies have highlighted promising candidates that either increase the effectiveness of the host to kill *M. tuberculosis* or reduce the destructive nature of an overexuberant host response, thus enhancing the effectiveness of pathogen-directed chemotherapy (16, 43–45). These approaches include arachidonic acid pathway modulators, NSAIDs (46), phosphodiesterase inhibitors (47), tyrosine kinase inhibitors (e.g., imatinib) (48, 49), antidiabetic drugs (e.g., Metformin) (50), and statins (51). Arachidonic acid pathway modulators provide a delicate balance in eicosanoid levels, enhancing *M. tuberculosis* control. NSAIDs interrupt the formation of proinflammatory and immunosuppressive mediators, such as prostaglandins and leukotrienes. Phosphodiesterase inhibitors reduce inflammation by increasing intracellular cAMP. Tyrosine kinase inhibitors are reported to reduce bacillary burden by promoting myelopoiesis, phagosome maturation, acidification, and autophagy. The immunomodulatory effects of antidiabetic drugs promote macrophage autophagy via the AMP kinase/mTOR loop. Statins have displayed control of lipid levels by targeting HMG-CoA reductase.

### M.

*tuberculosis* has strong adjuvant properties and promotes a robust Th1 response, resulting in chronic, local granulomatous inflammation (52, 53). That *M. tuberculosis* is deliberately immunogenic is counterintuitive, as the resulting response could eliminate the pathogen (53). Hence, *M. tuberculosis* invokes novel local mechanisms to potentiate its survival in the face of this immune stress to complete its life cycle, e.g., modulating TCR signaling (54) in a TLR-dependent manner (55, 56), phagolysosomal fusion (57), apoptosis (58), and IFN-γ (59) or TNF-α signaling (60). IDO catabolizes Trp (61) to starve pathogens of an essential amino acid (62). This strategy is, however, ineffective in restricting *M. tuberculosis*, which can synthesize Trp de novo (26). IDO blocks T cell proliferation downstream of IFN-γ, as Trp is essential for rapidly dividing effector cells (62). By reducing local Trp levels, IDO inhibits Th1 functions and generates Tregs, causing immunosuppression. This regulatory role of IDO is well studied in the immune escape of cancers and pregnancy, associated with poor prognosis (63), and linked to bacteremia (64). A decade ago we discovered that the expression of IDO is induced to very high levels in macaque TB granulomas (10, 14). Abundant data in both animal models as well as human samples since then strongly suggest that IDO is a key molecule that governs immunosuppression in the granuloma. The expression of IDO is highly induced in murine or primate macrophages (16), B6 (15) or Kramnik (16) mice, and macaques (16) upon *M. tuberculosis* infection. IDO expression is lowered in active TB (ATB) animals on chemotherapy or during nonpathogenic infection, is not induced in LTBI, and correlates with *M. tuberculosis* burden (16). IDO is expressed exclusively on myeloid cells in the inner ring of the granuloma (16). Single-cell RNA-Seq revealed that the majority of IDO transcript expression takes place on IFN-responsive, inflammatory, interstitial macrophages (4), as well as on immunosuppressive MDSCs (17), in the lungs of *M. tuberculosis*–infected macaques. IDO expression is not just a feature of TB granulomas in animal models. Single cell–resolution multiplexed ion beam imaging–TOF has revealed that IDO is one of the most highly expressed proteins in granulomas derived from human patients with TB (7). Furthermore, products of IDO-mediated Trp catabolism are detected in the plasma, sera, and urine of patients with ATB, including MDR-TB as well as TB/HIV, in cohorts from various regions of the world (18–20), correlating with prognosis and inversely with treatment (65). Inhibition of IDO signaling in *M. tuberculosis*–infected macaques improved clinical signs, bacterial burden, and lung pathology as a function of inhibition of IDO enzymatic activity (16). Taken together, these results from animal models of TB as well as patients unequivocally suggest that inhibition of IDO adjunctive to anti-TB chemotherapy is a viable HDT for patients with TB.

In the current study, we designed experiments to directly address if the addition of D1MT, an IDO inhibitor, adjunctive to TB chemotherapy improves the clearance of *M. tuberculosis* from the lungs of infected RMs and lowers the risk of TB disease. RMs closely represent several aspects of human TB, including ATB disease with high bacterial loads and pathology in the lungs, dissemination of *M. tuberculosis* to extrathoracic regions, and systemic inflammation; LTBI characterized by a lack of overt disease by microbiologic or radiologic measures but with immunological response to *M. tuberculosis* antigens; and HIV coinfection–mediated reactivation TB (31, 32, 35). Our model has also been utilized to study vaccine efficacy and mechanisms of protection as well as modeling antiretroviral (33, 34) and TB (66) therapies. We expose RMs to infectious aerosols of *M. tuberculosis*, thus mimicking the natural route of infection in humans. There are 3 typical models in our lab, where RMs are exposed to *M. tuberculosis* strain CDC1551, which has somewhat lower pathogenicity than the Erdman strain (67). RMs infected with 100–200 CFU of *M. tuberculosis* CDC1551 invariably develop ATB with a 100% progression to euthanasia within 3 months of infection. On the other hand, RMs exposed to very low doses of *M. tuberculosis* (~5–10 CFU) largely develop asymptomatic LTBI (33, 34). Exposure of RMs to approximately 25 CFU *M. tuberculosis* results in some RMs developing disease and others exhibiting control of infection (11). Treatment of nonhuman primates infected with drug-sensitive *M. tuberculosis* with frontline anti-TB chemotherapeutic regimen isoniazid + rifampin + pyrazinamide + ethambutol sterilizes lungs in macaques (68). To study the effectiveness of D1MT as an anti-TB HDT adjunctive to chemotherapy, we decided to develop a model of suboptimal anti-TB chemotherapy. Since HDTs are most needed in populations with drug-resistant TB, most such cases involve resistance to isoniazid (H), and moxifloxacin is used to replace it, we treated 2 groups of *M. tuberculosis*–infected (~25 CFU) animals with ME. Human equivalent doses of this regimen controlled *M. tuberculosis* infection but did not result in complete sterilization. This allowed us to study the impact of including D1MT as an adjunctive therapy in the second of the 2 treatment groups. Since IDO is a checkpoint inhibitor, modulation of its activity could lead to overexuberant immune responses and an uncontrolled pathology. We have earlier shown in a model of ATB that 4 to 5 weeks of treatment with D1MT is sufficient to reorganize the granuloma, modulate immune responses, and effect a reduction of *M. tuberculosis* burdens in RMs. We therefore used a treatment plan where 1 group was treated with ME at a time when many infected animals exhibited ATB (ME treatment group). The other group was similarly treated with ME but was only treated with D1MT for the initial 4 weeks (ME/D1MT treatment group). Our results show that while suboptimal therapy with ME reduces disease measures, inclusion of D1MT for only 4 weeks at the initiation of chemotherapy further enhances sterilization of *M. tuberculosis* while substantially improving T cell responses.

The results from our current study preclinically establish IDO inhibition, using D1MT, an approved, safe molecule currently in clinical trials, as a leading HDT strategy for TB. We have now shown its effectiveness in controlling progression of *M. tuberculosis* infection in 2 macaque studies, one in the setting of ATB and another in the setting of controlled progression and adjunctive to treatment. Further studies are necessary to elucidate if the mechanisms by which inhibition of IDO results in control of *M. tuberculosis* infection in both instances are shared and involve greater granuloma performance due to increased access of T cells to lesion core regions. It may also be important to further test the effectiveness of IDO inhibition in a different species of macaque, e.g., the cynomolgus macaque species, and in the setting of HIV coinfection. It would also be useful to test the effectiveness of D1MT in improving granuloma performance in the setting of LTBI. Fast-tracking this and other novel HDT strategies for TB in the clinical space may significantly improve treatment of TB.

## Methods

### Animals, infection, sampling, and euthanasia.

This study included 18 Indian origin RMs (*Macaca mulatta*) from 2 studies. Data were included from our recently completed studies, wherein specific pathogen–free, mycobacteria-naive Indian origin RMs were enrolled to the protocol after being obtained from a colony maintained at the Tulane National Primate Research Center (TNPRC, *n* = 9), the Southwest National Primate Research Center (SNPRC) (*n* = 6), or the Caribbean Primate Research Center (CPRC, *n* = 3) (Supplemental Table 1). All macaques were infected with an intermediate dose of approximately 25–50 CFU *M*. *tuberculosis* CDC1551 (BEI Resources, catalog NR13649) via aerosol as described before (14, 29–31). Tuberculin skin test was performed at weeks 3 and 5 after *M. tuberculosis* infection to confirm the infection. RMs were monitored for CRP, percentage changes in body weight and body temperature, and CBC weekly through the study period. A total of 12 macaques were then treated with the ME (M, 10 mg/kg; E, 20 mg/kg) regimen, beginning week 7 postinfection, for 12 weeks. A total of 6 of these RMs were also treated with D1MT daily (45 mg/kg, Sigma-Aldrich), as described earlier, for 4 weeks (week 7–11). This group that was also treated with D1MT is referred to as the ME/D1MT group while the group that only received TB chemotherapy is referred to as the ME group. A total of 6 RMs remained naive of all treatments during the protocol. The study demographics are presented in Supplemental Table 1. The assignment of macaques into 3 experimental groups was as follows. The D1MT/ME group included 2 macaques obtained from the TNPRC, 2 from the SNPRC, and 1 from the CPRC. The ME-only group included 2 macaques each from the SNPRC, TNPRC, and CPRC. The untreated group included 4 macaques from the TNPRC and 1 each from the SNPRC and CPRC. The macaque that received partial treatment was obtained from the TNPRC (please see Supplemental Table 1 for more details). This macaque was initially included in the ME/D1MT treatment group but rapidly progressed to ATB, and a decision was made by the veterinarian to euthanize it 2 days later.

### PET/CT.

Three sequential PET/CT scans were performed, using Mediso’s LFER150 PET/CT scanner, at 6, 12, and 18/19 weeks after *M. tuberculosis* infection with the last scan prior to necropsy. PET/CT scanning was essentially performed as described earlier (66). Briefly, we performed FDG PET/CT scans for each anesthetized macaque using the breath-hold technique (69, 70). Animals were anesthetized and intubated under supervision of a veterinarian as per approved IACUC protocols. All the animals received an intravenous injection of 5 mCi of FDG (71) in the right arm, procured from Cardinal Health radio pharmacy. Single- and a double–field of view CT scans were performed using breath-hold as described (72). The single–field of view (single-FOV) CT scan was performed with bread-hold as described previously (34) to obtain a clear reconstructed image of the lung; the 2-FOV scan was used for the reconstruction of the PET as the material map. Two FOV PET scans were acquired after a 45-minute FDG uptake period. Images were visualized using Interview Fusion 3.03 (Mediso) and reconstructed using Nucline nanoScan LFER 1.07 (Mediso) with parameters as described (73). 3D image analysis was performed using VivoQuant 4.0 (Invicro) (74) to calculate the SUV in the *M. tuberculosis* lesions observed in the lung.

### Microbiological evaluation.

Mycobacterial burden in BAL was measured throughout the study period as previously described (32). Viable *M. tuberculosis* burden was also measured at necropsy in BAL, lung, spleen, bronchial lymph node, mesenteric lymph node, liver, kidney, and individual granulomas collected at necropsy from each macaque (32, 75).

### Pathology evaluation.

This was performed as previously described (34). Briefly, the RMs were anesthetized for necropsy, and lung lobes, spleen, liver, kidney, bronchial lymph nodes, BAL, and blood were collected. Tissues were fixed in 10% neutral-buffered formalin, paraffin-embedded, sectioned at 5 μm thickness, and stained with H&E using standard methods. Stereology scores were prepared by a board-certified veterinary pathologist based on the percentage of multiple lung tissue sections affected.

### Confocal microscopy.

To validate various findings, multilabel immunohistochemistry was performed on *M. tuberculosis*–infected and *M. tuberculosis*–infected, ME- and ME/D1MT-treated RM lungs at necropsy as described (74). The lung sections were stained for CD68 (macrophages) and IDO-1. DAPI was used for nuclear staining. The slides were scanned using Zeiss Axio Scan Z1, and quantification was done using HALO software (Indica Labs). Kyn staining was performed on BAL cells from pretreatment and post–D1MT treatment time points, to quantify it before and after IDO inhibition. Briefly, BAL cells collected biweekly were concentrated on a slide in monolayer using Cytospin 4 centrifuge at 1,000*g*, 5 minutes, room temperature (Thermo Fisher Scientific). Images were captured using Zeiss LSM-800 confocal microscope, and ImageJ (Fiji) was utilized to quantify Kyn-positive cells in BAL. Antibodies used in immunofluorescence and immunohistochemistry are listed in Supplemental Table 2.

### qRT-PCR.

RNA was isolated from lung (obtained at endpoint) and BAL cells (from before and after D1MT treatment time point) using Direct-zol RNA Miniprep Kit (Zymo Research, catalog R2051) and quantified with Quant iT RNA HS Assay Kit (Molecular Probes, Thermo Fisher Scientific, catalog Q32852). Subsequently, RNA samples were reverse-transcribed to cDNA by utilizing iScript Advanced cDNA Synthesis Kit (Bio-Rad, catalog 1725038). cDNA obtained in this step was employed for measuring the levels of IDO1, IDO2, IFN-γ, and IFN-β1 genes by using TaqMan Gene Expression Assays (76) designed for RMs (Applied Biosystems, Thermo Fisher Scientific) with the following assay IDs: Rh02841203_m1 (IDO1), Rh04390839_m1 (IDO2), Rh02621721_m1 (IFN-γ), Rh02621721_m1 (IFN-γ), Rh03648734_s1 (IFN-β1), and Rh02621745_g1 (GAPDH). Fold expression of these target genes was computed relative to GAPDH using ΔΔCt method (or 2^-ΔΔCt^).

### Flow cytometry.

High-parameter flow cytometry was performed on BAL cells and PBMCs at preinfection, at pretreatment, during treatment, at posttreatment, and at necropsy as previously described (17, 34, 66, 74, 77). The single cells prepared from lung, BAL, PBMCs, and other tissues were stained with surface and intracellular markers to study T cell phenotypes. Tissues obtained at necropsy were digested using Liberase and DNase (both Sigma-Aldrich), filtered, and subjected to RBC lysis (ACK Lysis Buffer, Gibco). The cells were then counted and used for staining for flow cytometry. The cells were first stained with extracellular/surface antibodies: CD3, CD4, CD8, CD45, CD28, and CD95 for 25 minutes at room temperature, followed by the Fixable Viability Stain 575V (BD Biosciences). The cells were then fixed and permeabilized using Fixation/Permeabilization Kit (BD Biosciences) for 30 minutes at 4°C. Subsequently, the cells were stained with intracellular antibody (Ki67) to study the T cell proliferation. Cells were then washed and acquired on a BD FACSSymphony flow cytometer. Analysis was performed using FlowJo (v10.5.3) using previously published gating strategies (Supplemental Figure 4) (30, 32–34). The details of antibodies used in flow cytometry experiments are provided (Supplemental Table 3).

### Statistics.

Statistical analysis was performed using 1-way ANOVA with Tukey’s correction, 2-way ANOVA with Tukey’s multiple-comparison test, contingency χ^2^ (and Fisher’s exact) test, and Kruskal-Wallis test as applicable using GraphPad Prism (version 9). A *P* value of less than 0.05 was considered statistically significant. Data are represented as mean or mean ± SEM, as applicable.

### Study approval.

All infected macaques were housed under Animal Biosafety Level 3 facilities at the SNPRC, Texas Biomedical Research Institute, where they were treated according the standards recommended by the Association for Assessment and Accreditation of Laboratory Animal Care International and the NIH *Guide for the Care and Use of Laboratory Animals* (National Academies Press, 2011). The study procedures were approved by the Animal Care and Use Committee of the Texas Biomedical Research Institute.

## Author contributions

SM designed the study. BS, CM, DKS, RAE, RS, GA, XA, and SRG researched. BS, CM, DKS, and XA analyzed data. VS, OG, EJD, and SHU provided veterinary medicine and pathology analysis. SM and DK provided funding. SM wrote the initial draft. SM, XA, DK, BS, CM, and DKS edited the manuscript. All authors contributed to the manuscript.

## Supplementary Material

Supplemental data

Supplemental table 1

Supplemental table 2

Supplemental table 3

## Version 1 01/24/2023

Electronic publication
