# Supplementary material for: Inhibition of indoleamine dioxygenase leads to better control of tuberculosis adjunctive to chemotherapy
Source: JCI Insight. 2023 Jan 24;8(2):e163101. doi: 10.1172/jci.insight.163101 (PMC9977315; doi:10.1172/jci.insight.163101)
Supplement: Supplemental data [file jciinsight-8-163101-s100.pdf]

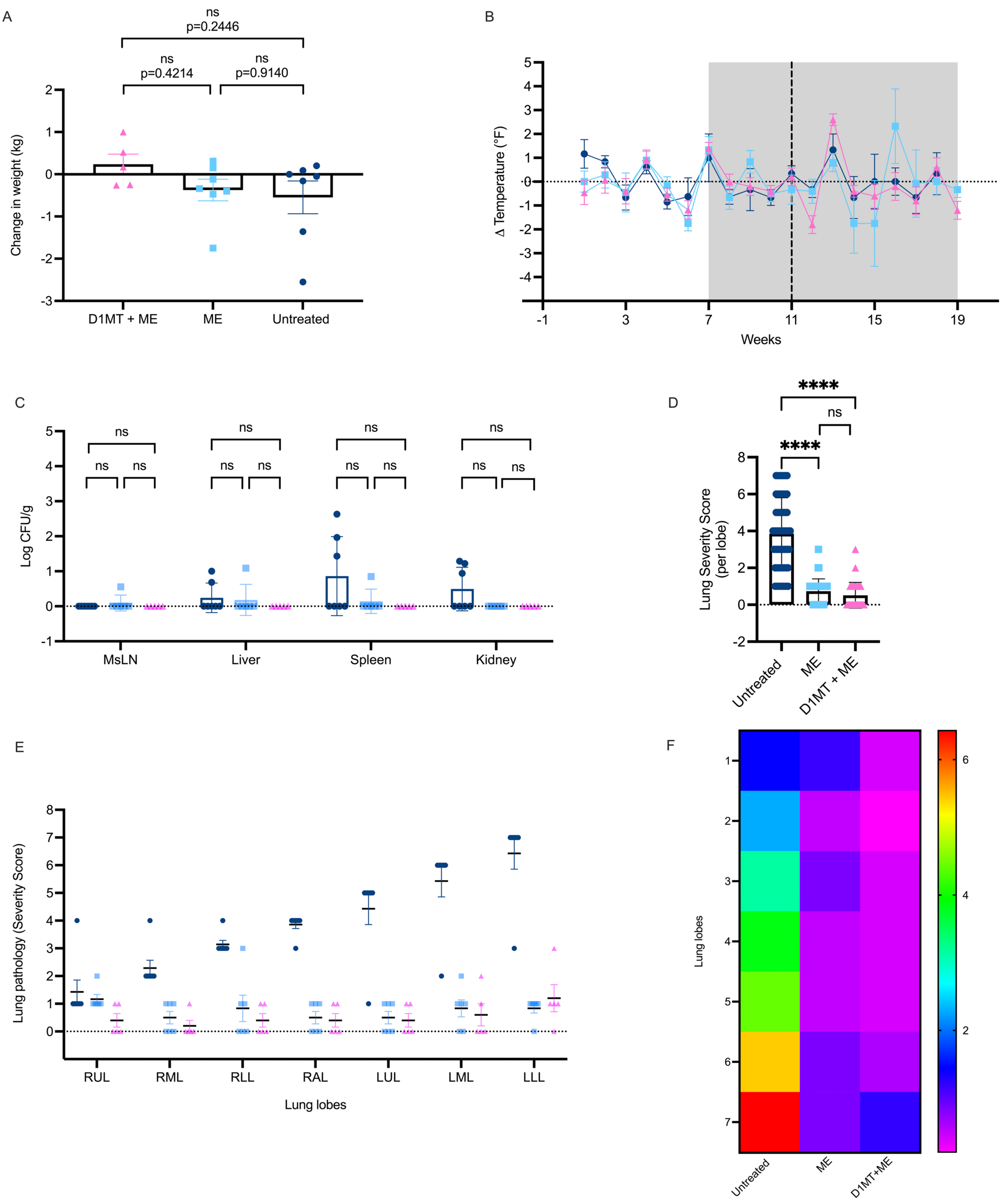

Supplement figure 1:

TB disease attributes were improved in treated macaques. **(A)** Figure showing the change in weight (kg) at the endpoint in macaques belonging to the three study groups. **(B)** Graphical representation of change in temperature at several timepoints of the study. Grey area represents the whole treatment period; whereas black dotted line shows end of D1MT treatment timepoint. **(C)** Graph showing Log Mtb CFU/g of Mesenteric lymph node (MsLN), liver, spleen and kidney obtained from untreated, ME only and D1MT+ME treated groups. Gross pathology of various lung lobes in the study groups were assessed for overall pathology/ severity by the board-certified pathologists in a blinded manner. Severity score in the all the lung lobes of untreated, ME only and D1MT+ME treated macaques where each dot/ square/ triangle represents a different lobe **(D)**. Graph showing the severity score in individual lung lobes- right upper lung (RUL), right middle lung (RML), right lower lung (RLL), right accessory lobe (RAL), left upper lobe (LUL, left middle lobe (LML) and left lower lobe (LLL) **(E)**. Heat map depicting the lung pathology with highest scores present in untreated group **(F)**. P values are indicated above the plots as obtained from one-way ANOVA **(A)** and **(D)** and two-way ANOVA **(C)** and **(E)** with Tukey's multiple comparison test. ****P <0.0001. Data are represented as mean ± SEM.

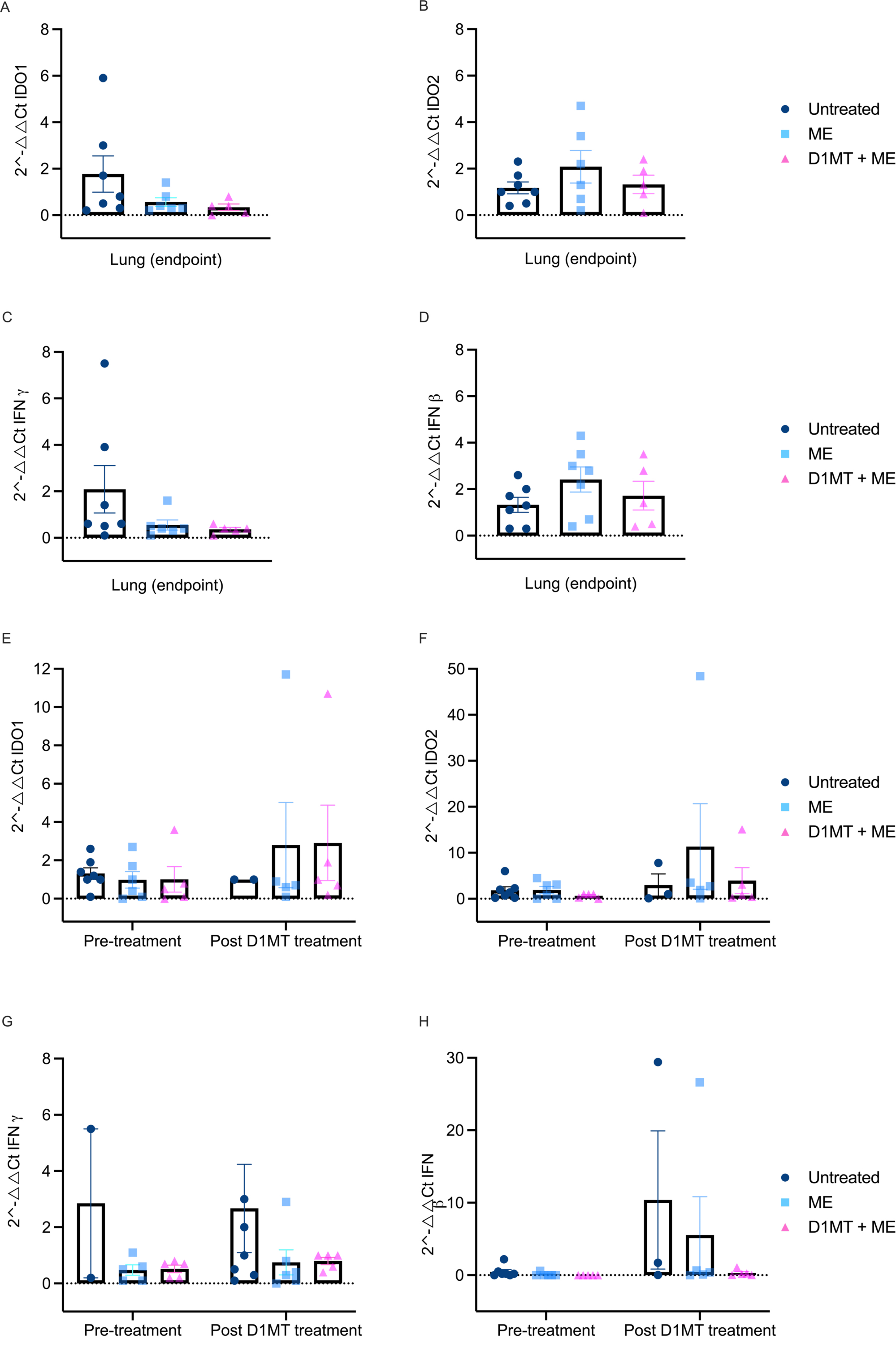

Supplement figure 2:

Quantification of IDO1, IDO2, IFNγ and IFN*β* by qRT-PCR in lung and BAL. RNA from lung obtained at endpoint and BAL cells from week 7 (pre D1MT treatment) and week 11 (post D1MT treatment) were isolated and quantified using Qubit. RNA was then reverse transcribed to form cDNA, which was then used to perform quantitative RT-PCR. Fold gene expressions were then quantified using delta-delta Ct method. Graphs depicting fold gene expression [2^-(ΔΔCt)] of IDO1 (**A**), IDO2 (**B**), IFN**γ** (**C**) and IFN*β* (**D**) in lungs and IDO1 (**E**), IDO2 (**F**), IFN**γ** (**G**) and IFN*β* (**H**) in BAL before and after D1MT treatment. No significant differences were observed in the levels of these genes among the three study groups. Analysis was done using one-way ANOVA (**A-D**) and two-way ANOVA (**E-H**) with Tukey's multiple comparison test. Data are represented as mean ± SEM.

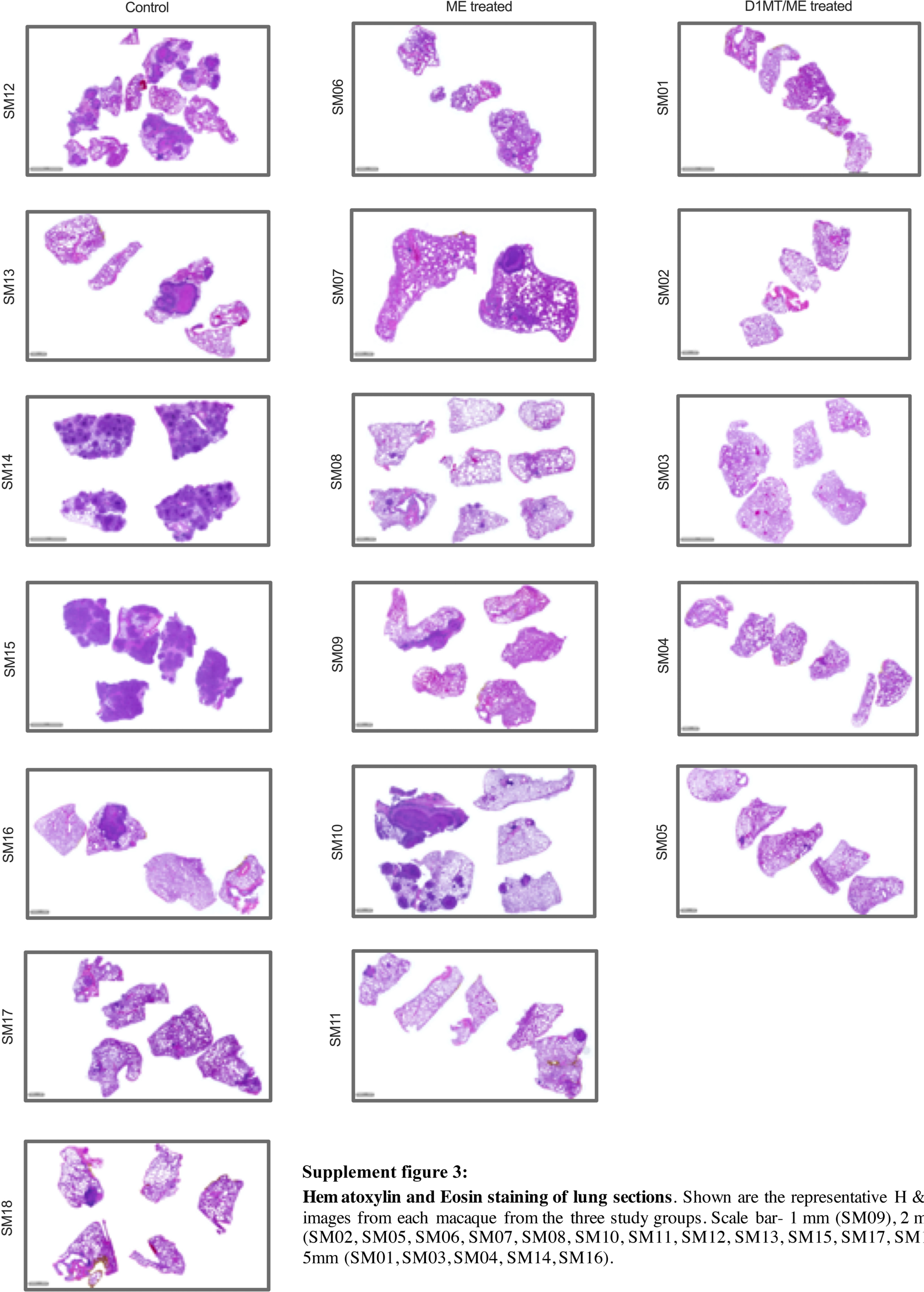

Supplement figure 3:

Hem atoxylin and Eosin staining of lung sections. Shown are the representative H & E images from each macaque from the three study groups. Scale bar- 1 mm (SM09), 2 mm (SM02, SM05, SM06, SM07, SM08, SM10, SM11, SM12, SM13, SM15, SM17, SM18) 5mm (SM01, SM03, SM04, SM14, SM16).

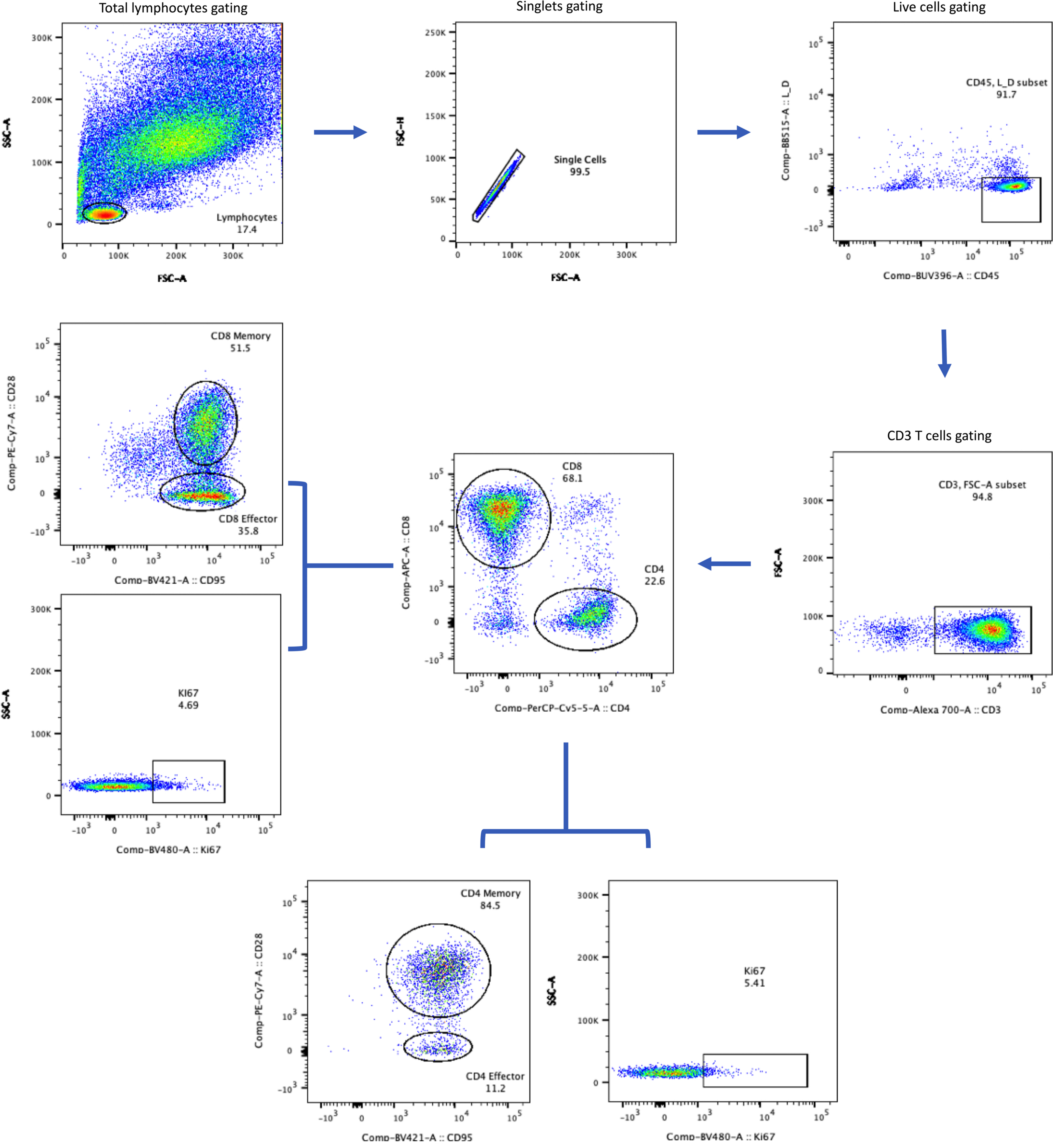

Supplement figure 4:

Flow cytometry gating strategy for analysis of T-cell panel. First total lymphocytes were gated from the FSC-A vs SSC-A plot. Total lymphocytes were then plotted FSC-A vs FSC-H to gate out doublets. The single cells were then gated for CD45 and Live/dead. CD45+ and live/dead- cells were gated for CD3. Total CD3 cells were plotted for CD4 and CD8. Subsequently, CD4/CD8 cells were then plotted for CD95 vs CD28, to gate for the CD4/8 effector cells (CD95$^{high/+}$ CD28$^{low/-}$) and CD4/8 memory cells (CD95$^{high/+}$ CD28$^{high/+}$). Total CD4/8 cells were then plotted for Ki67 to gate CD4/8+ Ki67+ cells.

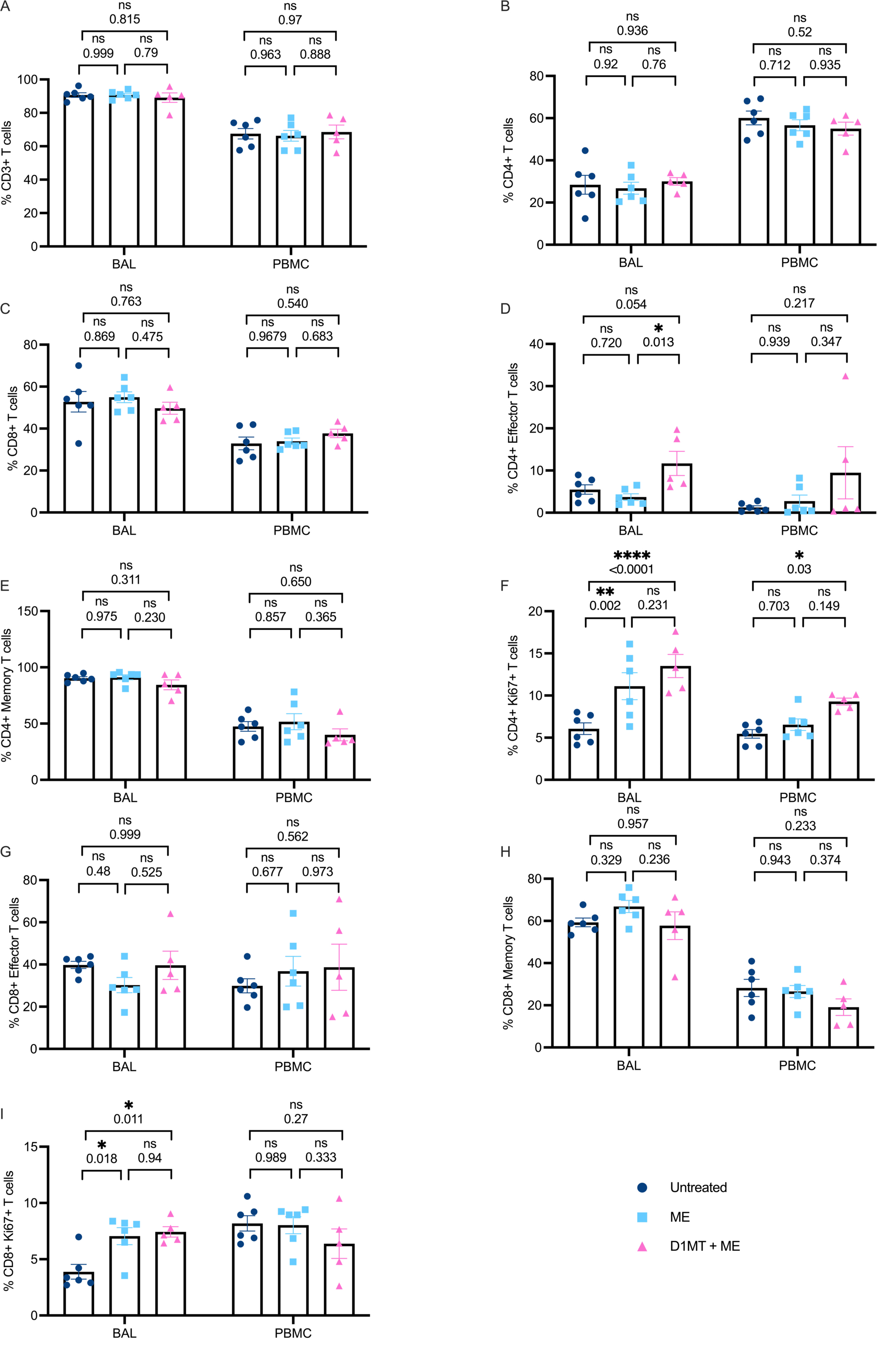

Supplem ent figure 5:

T-cell enum eration and function in BAL and PBMCs at the endpoint. BAL and blood at the endpoint were processed to obtain single cells, which were subsequently stained for T cells and other functional markers. (A-I) The graphs show the percentages of total CD3+ T cells (A), CD4+ T cells (B), CD8+ T cells (C), CD4+ Effector T cells (D), CD4+ Memory T cells (E), CD4+Ki67+ T cells (F), CD8+ Effector T cells (G), CD8+ Memory T cells (H) and CD8+Ki67+ T cells (I) in BAL and PBMCs at the endpoint. Significant increase in CD4 effector T-cells was observed in ME/D1MT treated animals as compared to ME alone group. We observed significantly high proliferative T-cells in both the treated group as compared to untreated macaques. *P* values are indicated above the plots as obtained from one-way ANOVA with Tukey's multiple comparison test. Data are represented as mean ± SEM.
